# The complete mitochondrial genome and phylogenetic analysis of *Littorina brevicula* (Gastropoda, Littorinidea)

**DOI:** 10.1080/23802359.2020.1772145

**Published:** 2020-06-02

**Authors:** Jie Bai, Yahong Guo, Jiantong Feng, Yingying Ye, Jiji Li, Chengrui Yan, Shuai Mao

**Affiliations:** National Engineering Research Center for Marine Aquaculture, Zhejiang Ocean University, Zhejiang, P.R. China

**Keywords:** *Littorina brevicula*, mitochondrial genome, Illumina sequencing, phylogenetic

## Abstract

The complete mitochondrial genome of the Littorina brevicula was determined in this study. The complete mitogenome (mtDNA) is 16,356 base pairs (bp) in length and contains 13 protein-coding genes, two rRNA genes, and 22 tRNA genes. The control region was divided into two parts. The overall base composition of the genome in descending order was 35.33%—T, 28.41%—A, 20.43%—C, and 15.81%—G. In 13 protein-coding genes, 12 genes start with ATG, except nad5 starts with ATT. For the stop codon, seven genes end with TAA, atp6, nad41, and nad3 end with TAG. Phylogenetic analysis indicated that *L. brevicula* is close to Naticidea family. This study first determined the complete mitochondrial genome of *L. brevicula*. It would be a supplement for the genetic analysis of *L. brevicula* and promote the phylogenetic of Littorinidea.

*Littorina brevicula* belongs to the family Littorinidea. The length of an adult shell varies between 11 mm and 23 mm, and its spawning season is from January to April (Son and Hong 1998). *Littorina brevicula* inhabits in the littoral fringe of the temperate coast of the northwestern Pacific (Reid 1996). *Littorina brevicula* is one of the most common snails, occurring in dense aggregations on rocky and boulder shores and on artificial constructions such as breakwaters, tetrapods, and slipways (Azuma and Chiba 2016). It is the main member of the biological community in the intertidal zone, the representative species in the lithofacies, with a strong resistance to environmental pressure (Vernberg and Vernberg 1972). However, the Littorinidea taxonomy is chaotic and the genetic information of *L. brevicula* is not clear.

Herein, it is the first time to determine the complete mitochondrial genome of *L. brevicula* in this study. The specimen of *L. brevicula* was collected from the coastal area of Dalian city, Liaoning province, China (121.6°E, 38.9°N) and identified by morphology and deposited in Zhejiang Ocean University (Accession number: LB20190611). The total DNA extraction was utilized the salting-out method (Aljanabi and Martinez 1997) with the muscle. Then, total genomic DNA was diluted to a final concentration of 60–80 ng/μl in 1 × TE buffer and stored at 4 °C in our laboratory at Zhejiang Ocean University for further analysis. The genomic DNA was prepared in 400 bp paired-end libraries. The Illumina HiSeq X Ten platform was used to perform the high-throughput sequence. All the data was available and enumerated to the Microsoft oneDrive database (https://1drv.ms/w/s!ArF1Al5lLW_VcGBYkaT0zHqhUe0?e=j3SVfv).

The complete mitochondrial genome of *L. brevicula* is 16,356 bp in length (GenBank accession number: MT362562). The complete mitochondrial genome has 13 protein-coding genes, two ribosomal RNA genes (rRNA), and 22 transfer RNA genes (tRNA). The nucleotide composition for *L. brevicula* is 28.41% of A, 35.33% of T, 20.43% of C, and 15.81% of G. In 13 protein-coding genes, only nad5 starts with ATT, the others start with ATG. For the stop codon, atp6, nad41, and nad3 end with TAG, nad1 end with TTG, nad6 ends with GCA, nad5 ends with TTA, other seven genes end with TAA. The 16S rRNA is 1396 bp between the tRNA-Val and tRNA-Leu2, and the 12S rRNA is 894 bp between the tRNA-Glu and tRNA-Val.

The phylogenetic tree (Figure 1) was constructed based on 13 protein-coding genes of *L. brevicula* and other 11 species using the Neighbour-joining method in the program Phylip (Felsenstein 1993). The result showed that *L. brevicula* is close to Naticidea family. We suggest that this result will further supplement the genome information in mitochondria of the family Littorinidea and facilitate the study on population genetic.

**Figure 1.**
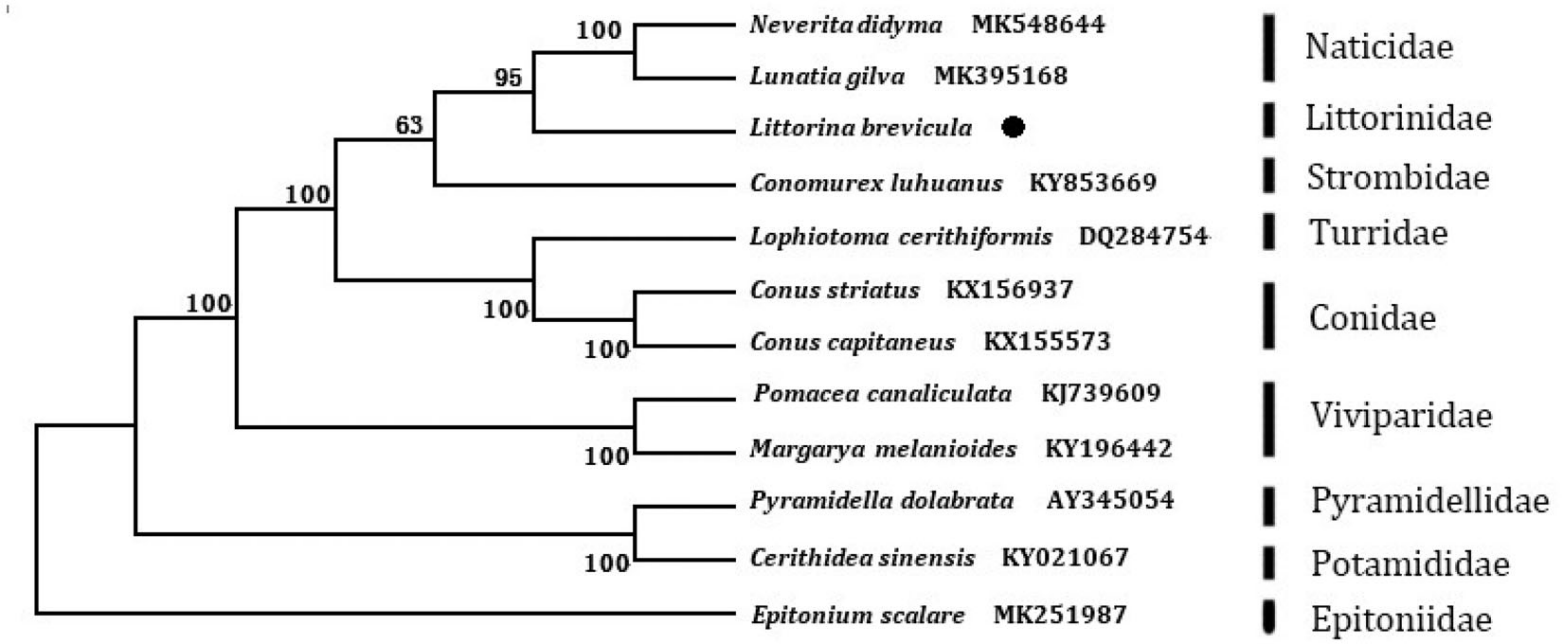
The NJ phylogenetic tree for *Littorina brevicula* and other species using 13 protein-coding genes.

## Data Availability

The data that support the findings of this study are openly available in Microsoft OneDrive at https://1drv.ms/w/s!ArF1Al5lLW_VcGBYkaT0zHqhUe0?e=j3SVfv; and in Genbank, reference number: MT362562.
